# A Randomized Clinical Trial Comparing Dubuisson Laparoscopic Lateral Suspension with Laparoscopic Sacropexy for Pelvic Organ Prolapse: Short-Term Results

**DOI:** 10.3390/jcm13051348

**Published:** 2024-02-27

**Authors:** Ewelina Malanowska-Jarema, Andrzej Starczewski, Mariia Melnyk, Dulce Oliveira, Matteo Balzarro, Emanuel Rubillota

**Affiliations:** 1 Department of Gynecology, Endocrinology and Gynecologic Oncology, Pomeranian Medical University, 70-204 Szczecin, Poland; e_malanowska1@tlen.pl (E.M.-J.); andrzejstarcz@tlen.pl (A.S.); 2 Institute of Science and Innovation in Mechanical and Industrial Engineering (INEGI), 4200-465 Porto, Portugal; doliveira@inegi.up.pt; 3 Department of Urology, Azienda Ospedaliera Universitaria Integrata Verona, 37126 Verona, Italy; matteo.balzarro@aovr.veneto.it (M.B.); emanuele.rubilotta@aovr.veneto (E.R.)

**Keywords:** dubuisson lateral suspension, uterine prolapse, minimally invasive surgery, pelvic organ prolapse, sacrocolpopexy

## Abstract

Background: Laparoscopic sacrocolpopexy (LSC) is the gold standard for the treatment of apical prolapse, although dissection of the promontory may be challenging. Laparoscopic lateral suspension (LLS) with mesh is an alternative technique for apical repair with similar anatomical and functional outcomes, according to recent studies. The purpose of this study was to compare these operative techniques. Methods: Women with uterine Pelvic Organ Prolapse Quantification (POP-Q) stage 2 were enrolled in this prospective study and were randomly allocated to the LLS or LSC group. At the 12-month follow-up, primary measures included both anatomical and functional outcomes. Perioperative parameters and complications were recorded. Results: A total of 93 women were randomized, 48 in the LLS group and 45 in the LSC group, with 2 women lost to follow-up in both groups. LSC anatomic success rates were 81.82% for the apical compartment and 95.22% for the anterior compartment. LLS anatomic success rates for the apical and anterior compartments were 90% and 92.30%, respectively. The mean operative time for LLS was 160.3 min, while for LSC it was 168.3 min. The mean blood loss was 100 mL in both procedures. Conversion to laparotomy was necessary in three women. Mesh erosion was not observed in any of the cases. In terms of the complication, Clavien–Dindo grade 1 was observed in two patients in the LLS group and a complication rated grade 3b was observed in one patient in LSC group. Conclusions: LLS is a good alternative to LSC, with promising anatomical and quality-of-life results.

## 1. Introduction

Pelvic organ prolapse (POP) is one of the most common causes of surgery in women due to its high incidence and effect on quality of life [1,2,3,4,5]. Apical defect rates range between 5 and 15%, and many procedures and materials have been proposed to improve the outcomes for the treatment of this compartment [6,7,8,9,10,11]. Laparoscopic sacrocolpopexy (LSC) is currently considered the gold standard surgery for apical prolapse [12,13,14,15,16,17,18,19,20,21]. However, few randomized clinical trials compare sacropexy with other surgical techniques used in the treatment of apical defects [22,23,24,25,26]. Dubuisson described a surgical technique to suspend the apical compartment: laparoscopic lateral suspension (LLS) [27,28]. As an alternative to LSC, lateral suspension does not require dissection in the area of the promontory, and thus does not pose a risk of major complications related to this surgical step [28,29].

The aim of our randomized trial was to compare the anatomical and functional effectiveness and the complication rates of these two procedures.

## 2. Material and Methods

This prospective randomized study included all women consecutively referred to our department from January 2018 to December 2021 with symptoms of a stage II or greater apical prolapse according to the Pelvic Organ Prolapse Quantification System (POP-Q), with or without anterior compartment prolapse. Informed consent was obtained from all patients. The experimental procedures were carried out in accordance with the principles of the Helsinki Declaration. This study was approved by the Ethics Committee on Clinical Studies of Pomeranian Medical University (KB-0012/27/17).

Exclusion criteria were previous urogynecological procedures (vaginal native tissue and mesh procedures, mesh abdominal fascia fixation, lateral repair and others); histologically confirmed cervical pathologies—CIN2/CIN3; dementia and other non-stable neurological diseases; associated posterior vaginal wall defects; and stress urinary incontinence grade II/III.

Objective evaluations were performed with the international POP-Q. Prolapse was assessed by maximum-effort Valsalva maneuver in the seated semi-lithotomy position. The evaluation included the type of defect (apical or apical plus anterior vaginal wall prolapse) and its severity according to POP-Q [30].

Follow-up was scheduled for 12 months after the surgery and performed by a skilled urogynecologist (EM). Objective cure for the apical prolapse was defined as POP-Q stage < II. Post micturition trans-vaginal ultrasonography was performed to assess the post-void residual urine (PVR).

Pelvic floor disorders, lower urinary tract symptoms and digestive symptoms were recorded in detail before surgery and at follow-up. The assessment was achieved by objective evaluation, medical interview and with the use of a validated questionnaire, the Pelvic Floor Distress Inventory Questionnaire (PFDI-20) [31]. All data were entered into a database and evaluated by one author (EM).

### 2.1. Surgical Technique

All the women underwent laparoscopic supracervical hysterectomies with or without concomitant prophylactic salpingo-oophorectomy (women over the age of 60) after reading and signing the informed consent form.

**Figure 1:**
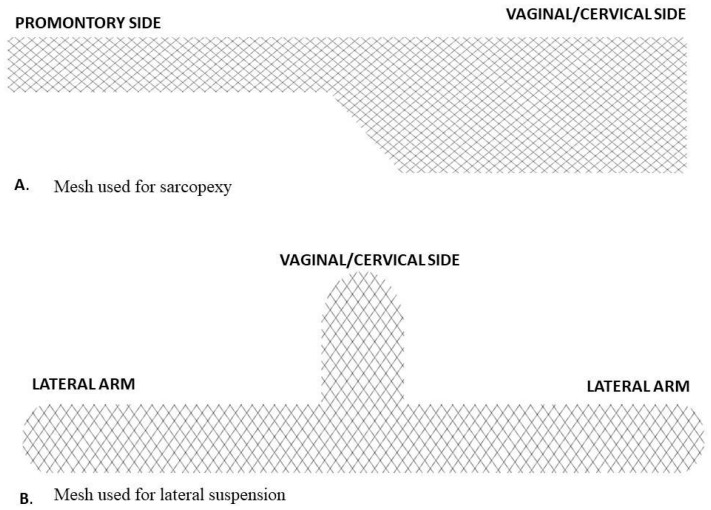
Mesh types used in this study.

Laparoscopic sacropexy was performed with single vaginal mesh (Figure 1A). Peritoneal incision and dissection started at the level of the promontory and was carefully extended along the rectosigmoid to the uterine cervix. The mesh was fixed to the anterior vaginal wall and cervix with four single non-absorbable sutures. The mesh was fixed to the promontory with two non-absorbable sutures (Ethibond 0). The peritoneum was then closed with a running suture (Vicryl 3-0).

A T-shaped polypropylene mesh was used for the lateral suspension (Figure 1B). The body of the mesh was fixed to the uterine cervix and the upper part of the anterior vaginal wall. The arms were introduced retroperitoneally towards lateral abdominal walls, alongside round ligaments. Both arms were attached laterally to the abdominal fascia without tension. Mesh peritonization was routinely performed.

After the prolapse reduction using the posterior blade of a speculum placed in the anterior vaginal fornix, the mesh was suspended tension-free in both procedures [32].

We did not perform any other concomitant pelvic reconstruction surgery for POP repair or urinary incontinence.

### 2.2. Power Calculation

Given a 2-year objective success rate of 76% for SP (sacropexy) and 89% for LS (lateral suspension), the sample size required to detect a 30% difference in success rates with a power of 80% and alpha 0.05 was 47 per group [33,34,35]. To allow for a drop of 15% and to ensure an adequately powered study, 100 participants were recruited.

### 2.3. Randomization and Blinding

**Figure 2:**
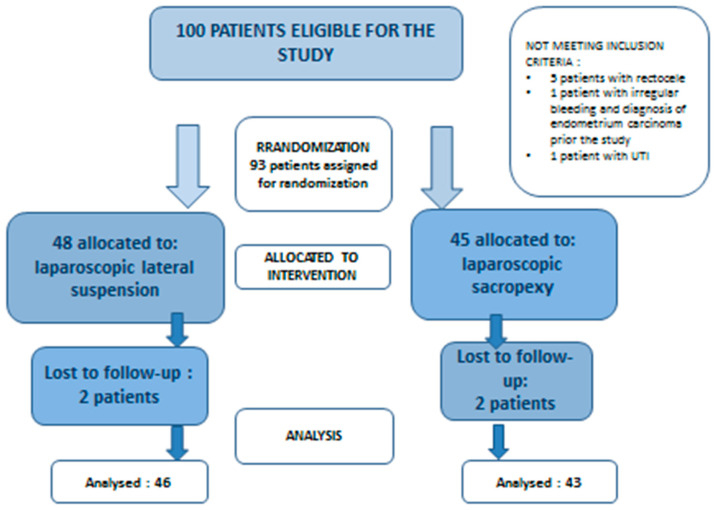
Two-armed, parallel study design.

Of the 100 patients identified, 7 did not meet the inclusion criteria. The remaining 93 patients were included: 48 underwent laparoscopic lateral suspension and 45 received laparoscopic sacrocolpopexy (Figure 2). Four patients were lost to follow-up. The total number of women who completed the 1-year follow-up was 89: 51.7% (46/89) for lateral cervicopexy and 48.3% (43/89) for sacrocolpopexy.

Participants were randomized to laparoscopic sacropexy or laparoscopic lateral suspension. The allocation sequence was created using a sealed envelope system, with a 1:1 ratio. At follow-up, the medical doctor was blinded to the group intervention allocation.

### 2.4. Patient Characteristics and Comparisons between Groups

The demographic characteristics and preoperative risk factors were similar in the two groups and are summarized in Table 1.

Preoperative characteristics did not differ between the two groups.

### 2.5. Statistical Evaluation

Based on the collected data, a database was created using Microsoft Excel^®^ 2013 (15.0.5589.1000) MSO (15.0.5589.1000) (32-bit), from Microsoft Office Standard 2013, Microsoft Corporation, manufacture code DG7GMGF0D7FX:0002. The data were statistically analyzed using Gretl software version 2017a. Quantitative variables are presented in the tables, giving medians, standard deviations, maximum and minimum values, and numbers in subgroups. Comparisons were made between LLS and LSC preoperatively and 12 months postoperatively; the *p* value was obtained using a *t*-test. The significance level was assumed to be *p* < 0.005.

## 3. Results

### 3.1. Evaluation of Anatomical and Functional Outcomes between Groups

All patients had significant stage 2 POP or greater in at least two of the three compartments (defined as 1 cm ≥ point C≥ −1 cm) before the surgery.

Clinical evaluation of pelvic organ support was assessed by Pelvic Organ Prolapse Quantification (POP-Q).

Anatomic cure was the first primary outcome and defined as POP-Q sites Aa, Ba, Ap, Bp and C less than −1 cm and POP-Q stage ≤ II.

A total of 93 women were randomized, 48 in the LLS group and 45 in the LSC group, with 2 women lost to follow-up in both groups.

By accepting as objective anatomical outcome POP-Q stage ≤ II, complete recovery in apical POP was found in 18 (81.82%) postoperative patients in the LSC group. In four (18.18%) patients, we observed recurrence of the primary stage of prolapse (Table 2). In the LLS group, complete recovery was found in 18 (90%) postoperative patients. In two (10%) patients, we observed recurrence of the primary stage of prolapse (Table 2). There was a clinically and statistically significant anatomic improvement in apical prolapses in both groups.

Patients with a mixed defect (cervical and anterior vaginal wall prolapse) had grade II cervical depression, so they were divided into two groups depending on the degree of anterior wall depression—stage II or III (Table 3).

A positive effect for the anterior compartment was found in 95.22% of patients after sacrocolpopexy vs. 92.30% after lateral suspension. This difference was not significant (*p* < 0.005). We did not observe any increased prevalence of the posterior compartment prolapse de novo in either group. Two symptomatic patients in the LLS group and one patient in the LSC group required posterior colporrhaphy after one year.

After 1 year, anatomically, there was a significant reduction in POP-Q points Aa, C and Ba as compared with the preoperative assessment (Table 4).

### 3.2. Operation Time

The differences in the mean operative time were not statistically significant. The mean operative time of laparoscopic sacrocolpopexy was 168.26 min (SD ± 37.37), and it was 160.33 min (SD ± 43.91) for lateral cervicopexy.

Both procedures were performed with concomitant laparoscopic supracervical hysterectomies. No other urogynecological procedures for POP or SUI (stress urinary incontinence) were performed.

### 3.3. Complications

Conversion to laparotomy was necessary in three women: two patients who underwent sacropexy and one in the lateral suspension group. These conversions were due to previous postoperative adhesions. Intraoperative complications included bladder injury (rated grade 1 using Clavien–Dindo classification) in two patients after laparoscopic lateral suspension surgery. These vesical lesions were immediately treated by laparoscopic suture repair. No other complications were recorded. In the sacrocolpopexy group, there was one complication rated grade 3b using Clavien–Dindo classification: one patient required a second-look laparoscopy due to severe lower back pain in the sacral area due to promontory fixation. We observed no vaginal mesh exposure in the 12-month follow-up.

### 3.4. Patient Satisfaction Evaluation

Patient satisfaction was assessed with the use of the validated questionnaire, PFDI-20.

There was a significant improvement in the main domains concerning prolapse, constipation and urinary symptoms in both groups (Table 5).

Analysis of quality of life using the validated questionnaire (PFDI-20) after both procedures showed a significant improvement, mainly in terms of bothersome symptoms (Table 6).

A total score of particular domains is depicted in Table 5. The evaluation of bothersome symptoms is shown in Table 6.

## 4. Discussion

The laparoscopic sacrocolpopexy is considered to be the gold standard for correcting an apical prolapse. Laparoscopic lateral suspension, first described by Dubuisson, is the most recent alternative surgical technique for apical POP.

To the best of our knowledge, this is the first randomized study demonstrating the comparison between LSC and LLS in anatomical, quality of life and symptomatological outcomes. In the presented study, major complications were not observed, and the surgical time was similar in both groups.

In our study, a high rate of anatomical objective success was found in both procedures, with no significant differences in either isolated apical or anterior vaginal wall prolapse. The overall LSC anatomic success rates were 81.82% for the apical compartment and 95.22% for the anterior compartment. LLS anatomic success rates for the apical compartment and for the anterior compartment were 90% and 92.30%, respectively, although Rubin et al. described a success rate of 75% for anterior defect after sacropexy and about 89% for lateral suspension [36].

At the beginning of the study, we excluded patients who had undergone previous urogynecological procedures, had associated posterior vaginal wall defects or had stress urinary incontinence grade II/III. The reason for this was that we aimed to obtain a homogenous group of patients. Such exclusion criteria do not reflect everyday life in urogynecology because anatomical defects occur that should sometimes be corrected simultaneously in one surgery [37,38]. However, for this study, more diverse groups could interfere with the results of the analysis. Therefore, we later included women with non-relevant additional defects to be treated in the study to avoid biases and to better assess how the vagina’s compartments will behave one year after the procedure.

In recent years, the standardized lateral suspension Dubuisson technique has been modified several times [28].

The risk of mesh-related complications increases with the size of the mesh [25,39]. In the so-called Mulayim technique, the authors suspended the vaginal vault, taking a double bite by using Mersilene tape [40]. This modified technique has the potential to be easier, more cost-efficient, less invasive and safer when compared with previously described methods where the typical polypropylene meshes were used.

Lateral suspension of the cervix follows the natural suspension of the uterus along the round ligaments, and in that case, the vaginal axis is preserved [41]. However, Rubin et al. suggested the need for concomitant treatment in case of muscle insufficiency [28]. The posterior compartment has to be evaluated and restored if necessary, as posterior levator ani plication may lead to a high risk of dyspareunia [42]. This step is still optional, and precise preoperative clinical evaluation should be carried out when there is an associated rectocele. In the case of anterior and posterior pelvic floor defects, the anatomical result of the posterior compartment is better when we associate posterior repair with a laparoscopic rectovaginal mesh or with a posterior colporrhaphy performed vaginally [43].

The first step of the LLS technique in our study consists of anterior dissection of the vesicovaginal space to the isthmus uteri. However, some authors are of the opinion that such mesh placement and ventralizing suspension have disadvantages for the posterior wall compartment. Recent studies showed that for the posterior compartment, a simple posterior colporrhaphy is the best option [44]. This was limited by the exclusion criteria; no posterior wall defects were included in this study.

In the modified laparoscopic lateral suspension technique with a five-arm mesh, the posterior compartment defect was repaired by suturing two posterior arms to the sacrouterine ligament, the posterior wall of the cervix and the posterior vaginal wall. The authors described how they achieved symmetrical anterior and posterior compartment suspension by elevating the posterior arms of mesh [45,46].

Primary in his technique, Dubuisson fixed the arms of the mesh to the lateral fascia. However, some cases of major pain due to lumbar plexus nerve entrapment were observed. In our short-term observations, we did not observe any lumbar plexus nerve entrapment.

What should be pointed out is the need to support the anterior compartment to avoid the occurrence of anterior vaginal wall prolapse using an appropriate technique. Dubuisson described dissection until 2 cm before the ureterovesical junction, and fixation of the anterior part of the “inverted T” mesh to the pubovesical fascia and the anterior wall of the vagina, as well as to the isthmus. However, some surgeons use Y-shaped mesh, preparing further down on the vaginal surface corresponding to a cystocele and rectocele. Some limit themselves to suspending the anterior or posterior vaginal wall with one strand, while others attach the mesh only to the cervical tissue [47,48,49,50,51,52,53].

It seems that stabilization of the vaginal apex (level 1) is an important component of operations to correct pelvic organ prolapse [53,54,55,56,57], and anatomical improvement of apical prolapse constitutes the keystone of repairing anterior vaginal wall defects [58].

It is debated whether the placement of mesh under the bladder may cause OAB symptoms. In our previous study [59], we found a negligible rate of de novo OAB symptoms in LLS (2.6%). The results presented in this study did not show a significant rate of OAB after sacropexy or lateral suspension.

Lower urinary tract injuries can occur during pelvic reconstructive surgery, including sacrocolpopexy. In the literature, reported injury rates range from 0.4% to 10.6% with laparoscopic sacrocolpopexy, 1.1% to 3.3% with abdominal sacrocolpopexy, and 2.3% to 10% with robotic sacrocolpopexy [60]. In our study, two bladder injuries occurred in the lateral suspension group.

In one of their first studies, Dubuisson et al. reported anterior vaginal mesh erosions in 4 (5.5%) patients out of a group of 73 patients [61]. We did not report any erosions in either of our groups. Also, the laparotomy conversion rate was similar for the two procedures. Thus, the risk of conversion was not a limitation for the Dubuisson technique. The advantage of the Dubuisson technique is that it avoids dissection at the level of the promontory and the associated risks of neurological, vascular, ureteral or other tissue lesions, which are commonly reported in obese women.

In addition to the principal operation of lateral suspension, Dubuisson et al. carried out the following procedures: Burch suspension, subtotal hysterectomy, bowel adhesiolysis, salpingo-oophorectomy or ovarian cystectomy, trachelectomy, transobturator tape (TOT), and ablation of mesh placed vaginally during a posterior intravaginal sling procedure. The mean operative time for such complex procedures was 208 min (SD 51 min; 95% CI 196–220 min) [61].

In the presented study, the mean operative time for laparoscopic sacrocolpopexy was 168.26 min (SD ± 37.37), and it was 160.33 min (SD ± 43.91) for lateral cervicopexy. Compared to other studies, our results were similar when taking into account concomitant supracervical hysterectomy [54,62,63]. Both procedures were performed with concomitant laparoscopic supracervical hysterectomies, which is the standard in our department. All the patients agreed to such proceedings. None of the patients preferred uterine preservation techniques. However, uterus preservation options should be discussed with every patient before surgery for POP [64].

Recent studies also show a benefit of uterine preservation by reducing the operating time [53,64]. It is assumed that lateral suspension with uterus preservation follows the natural ligament suspension of the uterus, providing a more physiological orientation of the vaginal axis [65,66]. Such “hysteropreservation” also avoids the risk of morcellation, which lengthens the operating time and can in some cases disseminate abnormal uterine tissue [66,67].

A standard sacrocolpopexy is generally carried out with a double prosthesis, during which an anterior and posterior mesh is generally placed. It is also hypothesized that vaginal erosions after LSC might be due to a different vascular supply of the posterior vaginal wall [67]. A preventive posterior correction must be weighed against the risk of complications. The primary goal of POP surgery care is to avoid hypercorrection and provide symptomatic relief without necessarily seeking perfect anatomical correction [67].

To our knowledge, there is no published report concerning a comparison of laparoscopic lateral suspension and sacrocolpopexy. The limitation of our study is the short time before follow-up with a single institution. Long-term follow-up is necessary to explore more information about the effects of these two procedures. Also, the laparoscopic procedures were performed by a single surgeon. In the future, surgical outcomes should be compared between novices and experienced surgeons, considering the duration of the surgery and the complication rate [68].

In summary, there were inconsistent results across studies, resulting in very low-quality evidence showing no difference in reoperation between mesh sacrocolpopexy and other techniques. Despite the large body of literature reviewed regarding adverse events, caution must be taken in interpreting these data, since there was much heterogeneity [68].

In conclusion, surgical correction of POP with LLS is a feasible alternative to sacrocolpopexy and can be performed with a concomitant subtotal hysterectomy. In our study, we presented comparable objective anatomical and patient satisfaction outcomes, as well as complication rates. Overall, our findings indicate that this technique is an effective approach to the treatment of apical POP in women and may be a safe alternative to sacrocolpopexy. We believe that our findings will promote the technique and be valuable for designing other trials with large sample sizes.

## 5. Conclusions

Based on our results, laparoscopic lateral suspension is a good alternative to laparoscopic sacrocolpopexy, with promising anatomical and quality-of-life results. However, more randomized controlled trials are necessary to establish the technique as an alternative to sacropexy in the treatment of apical prolapse [31].

## Figures and Tables

**Table 1:** Patients’ characteristics (mean, SD—standard deviation; LSC—laparoscopic sacrocolpopexy; LLS—laparoscopic lateral suspension).

Demographics	LSC *n* = 43	LLS *n* = 46	*p* Value
Age (years), mean (±SD)	58.14 (±8.28)	59.49 (±8.84)	*p* = 0.467
Age (years) of last period, mean (±SD)	50.44 (±3.76)	50.21 (±3.07)	*p* = 0.754
Number of patients after menopause (%)	41 (95.35)	42 (93.02)	*p* = 0.649
Number of vaginal deliveries, mean (±SD)	2.26 (±1.03)	2.42 (±1.18)	*p* = 0.497
Child’s birth weight, mean (±SD)	3743.72 (±402.07 g)	3625.81 (±345.82 g)	*p* = 0.149
Age at delivery, mean (±SD)	24.65 (±3.62)	24.37 (±4.39)	*p* = 0.749
BMI, mean (±SD)	26.59 (±3.73)	25.98 (±3.71)	*p* = 0.453

**Table 2:** POP-Q stage for apical prolapse before and at 12-month follow-up (LSC—laparoscopic sacrocolpopexy; LLS—laparoscopic lateral suspension; C—cervix).

POP-Q before LSC		POP-Q after LSC		POP-Q before LLS		POP-Q after LLS	
C	N%	C	N%	C	N%	C	N%
III	8 (36.6)	I	17 (77.27)	III	5 (25)	I	16 (80)
III	10 (45.45)	I	1 (4.55)	III	11 (55)	I	2 (10)
III	4 (18.18)	III	4 (18.18)	III	4 (20)	III	2 (10)
**TOTAL:** 22 (100)		22 (100)		20 (100)		20 (100)	

**Table 3:** POP-Q stage for apical and anterior vaginal wall prolapse before and at 12-month follow-up.

POP-Q before LSC			POP-Q after LSC			POP-Q before LLS			POP-Q after LLS		
A	C	N%	A	C	N%	A	C	N%	A	C	N%
III	II	16 (72.72)	I	I	12 (57.14)	III	II	17 (65.38)	I	I	14 (53.84)
			II	I	3 (14.28)				II	I	2 (7.69)
			III	I	1 (4.76)				III	III	1 (3.84)
II	II	5 (22.72)	I	I	5 (23.8)	II	II	9 (34.61)	I	I	7 (26.92)
									I	III	1 (3.84)
									II	II	1 (3.84)
**TOTAL**		21 (100)			2I (100)			26 (100)			26 (100)

LSC—laparoscopic sacropexy; LLS—laparoscopic lateral suspension; A—anterior vaginal wall; C—cervix.

**Table 4:** Anatomic changes in prolapse stage after surgery in patients undergoing LLS and LSC at 12-month follow-up (POP-Q points Aa, Ba, Ap, C and Bp above −1 cm).

LLS/LSC					
Postoperatively	Aa	Ba	Ap	Bp	C
Mean	1.50	1.39	1.59	1.98	5.36
SD	1.18	1.49	0.94	1.74	2.70
Chi-square	*p* > 0.005	*p* > 0.005	*p* > 0.005	*p* > 0.005	*p* > 0.005

**Table 5:** PFDI-20 domains (*p*—*p* value; Pelvic Organ Prolapse Distress Inventory 6 (POPDI-6), Colorectal–Anal Distress Inventory-8 (CRADI-8), and Urinary Distress Inventory 6 (UDI-6).

	Procedure	Preoperative (Mean)	Postoperative (Mean)	*p*	*p* Sacropexy/Lateral Suspension
POPDI6	LSC	52.91	6.69	<0.005	*p* = 0.460
	LLS	52.63	4.44	<0.005	
CRADI8	LSC	8.94	2.76	<0.005	*p* = 0.012
	LLS	11.13	6.65	0.043	
UDI6	LSC	44.96	10.66	<0.005	*p* = 0.077
	LLS	38.59	5.34	<0.005	
PFDI-20	LSC	106.81	20.11	<0.005	*p* = 0.491
	LLS	102.02	16.24	<0.005	

**Table 6:** Evaluation of symptoms using PFDI-20.

Symptoms	LSC *n* (%) 43		*p*	LLS *n* (%) 46		*p*	P LLS/LSC
	Preoperative	Postoperative		Preoperative	Postoperative		
Bulging	43 (100)	11 (25.58)	*p* < 0.005	42 (91.30)	4 (8.7)	*p* < 0.005	*p* > 0.005
Urinary urgency (wet)	24 (55.81)	7 (16.28)	*p* < 0.005	26 (56.52)	9 (19.56)	*p* < 0.005	*p* > 0.005
Urinary frequency	24 (55.81)	7 (16.28)	*p* < 0.005	29 (63.04)	10 (21.73)	*p* < 0.005	*p* > 0.005
OAB	16 (37.21)	4 (9.30)	*p* = 0.05	23 (50)	6 (13.04)	*p* = 0.005	*p* > 0.005
UI	11 (25.58)	7 (16.27)	*p* = 0.78	6 (13.04)	2 (4.35)	*p* = 0.08	*p* > 0.005
Constipation	27 (62.79)	12 (27.90)	*p* = 0,02	27 (58.69)	22 (47.82)	*p* = 0.187	*p* > 0.005

## Data Availability

Data is available on request due to the privacy of patients who participated in the research.
